# Hospital and Institutional News

**Published:** 1919-01-18

**Authors:** 


### HOSPITAL AND INSTITUTIONAL NEWS.

### ROYAL HOUSING SCHEME.

The Prince of Wales, as Lord of the Manor of Kennington and as Duke of Cornwall, has, like his predecessors taken a great interest in the Duchy of Cornwall estate in Kennington, where huge housing reforms have been carried out during the last few years. The estate has been a source of great income to Princes of Wales since the days of Edward III, who created his son, Edward the Black Prince, not only the first Prince of Wales, but also the first Duke of Cornwall. Indeed, the Kennington estate can date back to Saxon times as part of the Royal estates, for here was a palace at which both Hardicanute and Edward the Black Prince died. The estate has, however, fallen into bad repute as a residential district, through the fault of speculative builders, who between 1780 and 1830 obtained leases for the erection of dwellings. The great majority of these was shoddy like most of the buildings of the earlier part of the last century, and, as the leases began to run out, the dwellings became more and more dilapidated.

### THE VAST IMPROVEMENTS.

The first great batch of leases fell in about 1908, when the Council of the Prince of Wales took in hand a huge housing reform scheme. Slum areas were swept away, and on the site arose a model village. Commodious houses were provided at moderate rents, and special habitations for the old folk, wide streets and open spaces taking the place of the crowded and insanitary areas that existed previously. The war, unfortunately, stopped the great improvement that was going on in this part of South London, but with the cessation of hostilities the work is to be begun again, with the conversion of a number of empty houses into flats to be let at £20 a year. Unfortunately, similar conversions in the past have not been very encouraging, as the cost has been very little below that for erecting new houses. Difficulties have occurred similar to those that always arise when old houses are pulled about. Many may be verminous, and the usual expedients to cleanse the houses, such as fumigation and the removal of the woodwork, have failed to entirely remove the nuisance. In these circumstances, it is to be feared that little can be done at present, as the scarcity and high price of materials and labour, almost prohibit rebuilding at the present time. However, as soon as matters resume their normal condition the re-modelling of the estate of the Prince of Wales will be proceeded with.

### ARMY DOCTORS THANKED.

The pressing needs of the civil population, as represented by the voluntary hospitals and the Insurance Commissioners, has received the recognition of the Director-General, Army Medical Service, who intimates that it has been found necessary to demobilise a large number of R.A.M.C. officers at short notice. In announcing this decision the Director-General pays generous tribute to the personal sacrifices made by members of the profession in the desire to serve their country, and assures them of his "sincere gratitude for their valuable services." Officers and men of the various branches of the fighting Forces have severally received public thanks for their share in the great victory, and perhaps it was due to the doctors, whose services have been indispensable, and who have, as well, taken risks and paid heavy toll in life and suffering, that special recognition should be forthcoming from those in authority.

### MEDICAL OFFICERS' ORDER OF PRIORITY.

In determining the order of priority in which medical officers in the Army shall be demobilised the Ministry of National Service, which is the Department entrusted with this duty, has laid down two broad rules which shall govern release—(1) public need for the return of a particular medical officer, (2) the personal interests of the individual himself. In the first case application must be made to the appropriate central authority by the particular local body concerned. As regards release on private grounds, a form—M.N.S.(M.) 16, to be precise—is being issued to every medical man in the Army. Order of priority in this latter section will be decided on consideration of age, length of service, domestic services, and reasons connected with practice. In addition to these hard-and-fast rules, doctors can put forward claims for release on grounds other than those dealt with in the form referred to, but these must be put forward by way of a separate and special application. Generally speaking, the demobilisation now announced will be received with feelings of gladness by medical men, whose practices, to say the least, have not improved through their prolonged absence; by patients who have suffered long and uncomplainingly owing to men who are often friends and counsellors, as well as medical advisers, being away at the war; and by public bodies, who in the recent influenza epidemic received a lesson they are not likely to forget in a hurry on the imperative importance of possessing a capable and fully organised medical staff. Every scheme has its weak points, and no doubt this one will produce the usual crop of grumblers. The heaven-sent Public Department which can please every one has yet to be evolved. But no doubt doctors, who so often have to preach patience to others, will now show the public how to practise it.

### RELEASE OF DENTAL SURGEONS.

Along with the order concerning the release of doctors comes another, affecting members of the sister branch of dental surgery. In these cases the order of priority will be determined by the Ministry of National Service both in regard to surgeons who are serving with the Forces as dental officers and those who have found a place in the combatant ranks. The Dental Service Committee for England and Wales and for Scotland will advise the National Service Ministry on points which should determine priority in the numerous cases which will come up for consideration. The members of a long-suffering profession, by the way, would not be human if they did not discover cause for satisfaction in that part of the official announcement which states that "unregistered dentists, dental mechanics, and dental students will be dealt with by the Ministry of Labour according to the lines laid down for general demobilisation, and no application in respect of such men will by entertained by the Ministry of National Service." Parliament may be slow to move to give dental surgeons protection which common fairness seems to demand, but the Ministry of National Service clearly recognises a vast distinction between the registered and the unregistered practitioner of dentistry. Intentional or not, there is a pungency and a quiet effectiveness in the referring of "unregistered dentists, dental mechanics, and dental students" to be dealt with by the "Ministry of Labour." The hint will not be lost upon the dental surgeon, who, however, is by no means out to pose as vindictive or vengeful.

### ANTI-TOXIN INSTEAD OF SLEEP.

Men and women there are—they include some of the world's greatest workers—who laugh at long hours of labour and short periods of sleep. And, curiously enough, these sleepless people of dynamic energy as often as not live to a wonderfully ripe age. What is the secret of this immunity to the claims of sleep? Miss May Smith, lecturer in psychology at Oxford, is on its track. She propounds a theory, which at any rate has the merit of novelty, that the body in certain fortunate cases supplies naturally a "fatigue anti-toxin." The lady lecturer has been pursuing research to discover this new "philosopher's stone," but so far she has not got beyond the stage of interesting developments, though, like all true crusaders, she is hopeful, not to say sanguine, of ultimate triumph. Miss May Smith has clearly broken new ground, and provides scientists with something new for speculation. Our correspondence columns are open to the discussion of the scientific aspects of sleeplessness and its problems.

### TAMPERING WITH PRESCRIPTIONS.

The ordinary sane and healthy person usually regards a medical prescription with awe, sometimes amounting to veneration. He would as soon think of tampering with the King's currency as play tricks with the mysterious slips of paper he conveys to the pharmacist. Not so with the degenerate victim of the drug habit; nothing which stands in the way of the gratification of a diseased craving is sacred to him or her. In two recent cases which have come before the courts prescriptions were shown to have been tampered with or forged for the purpose of securing an illicit supply of a drug which can only be obtained on a medical man's order. These cases demonstrate the need for changes in the law which will not only secure punishment for the forgers, but go a long way to prevent the repetition of similar evils. Latin and an almost illegible scrawl are not the "barbed wire" defences they used to be.

### CORNWALL HOSPITAL SCHEME.

The governing body of the Royal Cornwall Infirmary, Truro, have under consideration a scheme for the development of the institution. The aim appears to be to provide for an increase of 50 beds, estimated to cost £4,000 per annum. As the income of the infirmary in 1916-17 was under £5,700, it will be gathered that the project is an ambitious one. It is desired to make the institution, in the full sense of the term, the county hospital and the centre of medical effort in Cornwall. The plans involve the construction of special departments for bacteriology, pathology, electrical treatment, massage, &c. The staff suggested is one physician, three or four surgeons, one ophthalmic surgeon, and one assistant physician and assistant surgeon. The appointments would be thrown open to medical men residing in the county. It is proposed to associate with the staff the county medical officer, the county tuberculosis officer, and the officer for venereal disease; to establish a consultative department and to institute discussions on interesting or obscure cases that may be sent in. So far, the medical profession, upon the support of whose members depends the success of the scheme, has not had full opportunity of expressing its opinion; and there seems to be a feeling in some quarters that the project might clash with the interests of local hospitals in the county. Others think that with the State on the threshold of action for the country in general this is an inauspicious time to launch out into an enterprise involving great changes and much money. The upshot of the Governor's meeting was a decision to postpone further action until the views of the medical profession have been obtained.

### WAR MEMORIALS AT BART'S AND GUY'S.

At both St. Bartholomew's and Guy's Hospitals the question of providing a fitting memorial to men who have fallen in the war is being considered. The two hospitals differ in their methods of dealing with the matter in this respect. While St. Bartholomew's asks for suggestions, Guy's asks for subscriptions, and is obtaining them to an encouraging extent. A strong committee has been formed at the great Borough hospital, and a circular letter has been issued suggesting that the memorial should take the form of (*a*) a permanent record in the hospital of the medical and dental men attached to the institution who have fallen in the war; (*b*) a fund to enable the sons of men who have fallen or suffered by the war to receive free or assisted education in the medical or dental schools; (*c*) a fund to enable the daughters of such men to receive free or assisted education as nurses or pupils in the special departments (such as the massage and light departments) which are open to women; and (*d*) an endowment fund for entrance scholarships to be open, in the first place, to the sons of old Guy's men. Such a liberal and useful scheme deserves, and is receiving, substantial support. We can suggest but one amendment, and that is that women should be admitted to the scheme providing for medical education.

### THE NEW MASTER OF THE NOSOCOMIA LODGE.

Sir Perceval Nairne completed his year of office as Master of the Nosocomia Lodge of Freemasons on the 10th instant, when he installed in the chair his successor, who is Mr. James McKay, the acting secretary of the Hospital for Sick Children. Mr. McKay, like most of his predecessors in the Master's Chair, is a well-known figure in voluntary hospital life in London. He is one of those assistant secretaries (such as was the late Mr. Walter Adams, of the Seamen's Hospital) whose experience and standing entitle him to rank with holders of the highest office of the service. Not for the first time does Mr. McKay act as secretary of the hospital in Great Ormond Street, for, after the death of Mr. Adrian Hope, and before the appointment of Mr. E. S. Darmady (now serving in the R.A.M.C.), quite a period elapsed during which he sat in the secretarial chair. We notice that Mr. G. A. Arnaudin, secretary of St. John's Hospital, Leicester Square, is the new Senior Warden, and Mr. A. H. Coughtrey, librarian at St. Bartholomew's, is the Junior Warden, while other offices are filled by notable hospital figures. The Nosocomia Lodge has been a success from its foundation, nearly ten years ago, and from the continued support it derives from hospital masons appears to have its immediate prosperity assured.

### HELP FOR PHARMACISTS.

A special fund has been inaugurated by the Pharmaceutical Society of Great Britain to assist pharmacists and assistants on their return from service with the Forces to civil life. "The War Auxiliary Benevolent Fund," as it is termed, now totals well over £10,000, of which amount Messrs. Boots provided the handsome sum of £3,000. Instead of paying this into the central fund, Sir Jesse Boot has given instructions that each district association of pharmacists shall be credited with a proportion based on the number of shops owned by the company in the area. The fund is not intended to supplant Government, but to augment such help and provide immediate assistance and facilities for students and graduates whose career has been interrupted by the call to arms. Assistance has already been granted in several cases, and the prompt and sympathetic treatment accorded them is greatly appreciated by the applicants.

### PHARMACIST EXAMINATION CHANGES.

The new by-laws of the Pharmaceutical Society, regarding conditions of training for the qualification of pharmacist, having been approved, will become effective, subject to the sanction of the Privy Council. The main alteration is that the present qualifying final examination is to be divided into two parts, and candidates failing to satisfy examiners in every subject may, under specified conditions, be credited with a "pass" in those subjects in which they have satisfied the examiners. The new conditions are expected to be a truer test of a candidate's knowledge of pharmacy and allied subjects, and will result, it is hoped, in less "cramming" of subjects purely for examination purposes.

### MOTOR AMBULANCES FOR THE PEOPLE.

The civil population are to benefit eventually by the generosity which has supplied with a lavish hand the British Red Cross Society and the Order of St. John with motor ambulances for war purposes. About 3,000 of these ambulances are in use in the war areas, and there is an additional large number at home. As part of the general work of demobilising the Red Cross forces, a scheme is afoot for using these ambulances in the service of the sick and injured throughout the country—to provide free transport in cases of illness or accident to working men or members of their families whom it may be necessary to convey to the civil hospitals. The general idea is, so far as possible, to return cars to the districts which originally presented them. Many places have long felt the need of motor ambulances for civil uses. In some fortunate towns the want has been supplied by the munificence of public-spirited citizens. This plan of the Joint War Committee will solve a serious problem in many districts if it comes to fruition.

### "PEOPLE'S LEAGUE OF HEALTH."

Miss Olga Nethersole, the actress, who for three years has been a nurse at the New End Military Hospital, Hampstead, has now founded a People's League of Health, which she hopes may grow into the educational branch of the future Ministry of Health. She believes that the mass of the people is as ignorant as it is poor, and the towns in which she went professionally have given her plenty of opportunity of judging, since she used to study the conditions of life. The League has a medical advisory council.

### EIGHT-HOUR DAY FOR GLASGOW NURSES.

Speaking at the Glasgow Victoria Infirmary, of which he is Chairman, Mr. William Gray said that the governors had decided, so soon as the extra accommodation required was obtainable, to reduce the hours of the nursing staff from sixty-five to fifty-six a week. He hoped that before many months had passed this might be obtainable, though for the eight-hours day a large addition to the present number of nurses would be required.

### EXTENSION OF ROYAL GWENT HOSPITAL.

The proposal to build a new wing to the Royal Gwent Hospital as a war memorial, though adopted tentatively, is one which should not be allowed to lapse. It is no use being second in the field. Few, if any, other proposals command general approval, and Sir Garrod Thomas's effort to enlist the support of public bodies, gave the proposal a good start, and in these matters a good start is a great advantage.

### PATIENTS' TELEGRAM TO THE KING.

The King's Fourth Annual Concert to military and civilian patients of the Great Northern General Hospital was held last week in the Islington Victoria Ward. Preb. C. J. Proctor, M.A., gave a brief address. It was resolved by those present that the following telegram should be despatched to His Majesty at Sandringham: "The patients, including one hundred and eighty wounded and discharged and disabled soldiers in the Great Northern Central Hospital, Holloway, humbly thank Your Majesty for the delightful entertainment graciously provided this evening, and offer their homage on the occasion." The series of illustrated lectures to the patients continues.

### STATISTICS OF AMERICAN RED CROSS WORK.

DETAILS of the Red Cross work under United States organisation during the war show that £4,426,000 was spent in France, £3,392,000 in Italy, £1,691,000 in Russia and Siberia, £457,000 in Switzerland, £543,000 in Rumania, £1,023,000 in Palestine, £2,166,000 in England, and £676,000 in Belgium. Total home service expenditure amounted to £1,200,000, with an equal amount spent on cantonment activities. Up to June 30 last the convalescent home service work had cost £300,000, with another £200,000 appropriated for it for the six months to the end of 1918. The Red Cross has approximately £2,600,000 worth of supplies in France for future use. Finished supplies valued at £11,800,000 made by Red Cross workers all over the United States have been turned out during the last seventeen months. It is estimated that during this time some eight million volunteer workers have turned out 253,196,000 surgical dressings and 22,225,000 articles for use in hospitals, as well as 15,000,000 knitted articles and garments for relief work.

### SHEFFIELD INSTITUTION FOR TUBERCULOSIS.

IT is a perturbing thought how much activity in civilian hospitals has been overlooked during war time. Opened three years ago, the beautiful Rivelin Valley home for the little tuberculous cripples of Sheffield is well worth a visit. Here may be found in operation those conservative methods of treatment introduced from the Continent and popularised at Alton with such success by Dr. Gauvain. Despite the handicap that celluloid for splints has been unprocurable during the war, and that the present time of year is unsuitable for heliotherapy, this institution serves well its 130 odd juvenile patients, whose rosy cheeks prove that rest in bed in the open air is the reverse of a debilitating regime. Their mental needs are provided for by visiting teachers, so that no interruption of education is entailed. The surroundings are exceptionally tasteful and artistic, and already the building is thoroughly harmonised with its rural and romantic site. Most of the woodwork is painted in two shades of blue, the rest in white enamel. There is a good *x*-ray installation, and a Hanan "künstliche Hohensonne" lamp for artificial heliotherapy. The foundation-stone was laid by the Duchess of Norfolk, whose husband was of course a large landowner in the capital town of Yorkshire.

### DOCTORS IN CHURCH.

FROM the pulpit of Leeds parish church Sir William Milligan gave an address on behalf of the improvement of Britain's health. It was confined to medical and administrative matters, and advocated a higher standard of living, the enemies of which were, he said, bad housing, excessive working days, low wages, restrictive output, intemperance, and shortage of hospitals. It is noteworthy that restrictive output was included, for Sir William Milligan evidently shares Lord Leverhulme's opinion that the cost of better houses, shorter hours, and higher wages must be paid for out of the increased production of the working classes. He also advocated municipal control of the milk trade, physical training, and the extension of allotments, and declared from the pulpit that the nation could afford to lose neither the legitimate nor the illegitimate child. Probably his hearers agreed with him. But is a high birth-rate an end in itself? Very few consider the matter. Yet the press of population was certainly one of the causes of the war.

### SUICIDE OF A HOSPITAL SURGEON.

THE happy record of the 3rd London General Hospital, Wandsworth, has been marred by the suicide of Dr. Ivor William Joynt, aged thirty-one, who had been a civil surgeon at the hospital since October 1916. He made no complaints, nor had any been made of him, and apparently he had no financial or other worries. It is true that he wrote to a woman friend at Bournemouth that he was "dead beat," but the phrase has not been explained, nor does the fact that he introduced the term "a neurotic" into a farewell letter to Colonel Bruce Porter throw much light upon the tragedy. Dr. R. C. Jewesbury, who made a post-mortem, said that death was due to narcotic poison, and that he found an abnormal thickening in the cerebral membranes.

### THE BETTER EDUCATED MEDICAL STUDENT.

ON this subject Dr. Waldo has made an interesting observation. In three cases he had held inquests on those dying under the influence of an anæsthetic, compared with seven such cases in 1917. This was the lowest number of deaths under anæsthesia recorded for the past nineteen years. The decrease was due to the greater care, skill, and discrimination now used in the administration of anæsthetics, together with the better education of the medical student.

### THIS WEEK'S DRUG MARKET.

BUSINESS still continues extremely quiet, buyers still holding off in the hopes of lower prices. Considerable quantities of various popular vegetable drugs have accumulated in London and will be offered for sale by public auction later this week. it will be of interest to note if, and how far, prices have receded since the last public sale some six weeks ago. Holders are certainly acting patiently and the trend of prices largely depends on whether they can be more patient than buyers. Since last report, most, if not all, the price changes have been in a lower direction. Aspirin tends downwards in price. Benzoic acid is quoted at a lower rate. The price of citric acid is not so firmly maintained. Saccharin continues to move downwards in price. Phenolphthalein and benzonaphthol are rather cheaper. The present quiet state of the market can only be due to the fact that buyers are putting off their purchases in the hope and expectation that values will recede much further.

